# *De novo* assembly of a genome-wide transcriptome map of *Vicia faba* (L.) for transfer cell research

**DOI:** 10.3389/fpls.2015.00217

**Published:** 2015-04-09

**Authors:** Kiruba S. Arun-Chinnappa, David W. McCurdy

**Affiliations:** Centre for Plant Science, School of Environmental and Life Sciences, The University of NewcastleNewcastle, NSW, Australia

**Keywords:** *Vicia faba*, transfer cell, wall ingrowths, *de novo* transcriptome assembly, transcription factors, RNA-Seq

## Abstract

*Vicia faba* (L.) is an important cool-season grain legume species used widely in agriculture but also in plant physiology research, particularly as an experimental model to study transfer cell (TC) development. TCs are specialized nutrient transport cells in plants, characterized by invaginated wall ingrowths with amplified plasma membrane surface area enriched with transporter proteins that facilitate nutrient transfer. Many TCs are formed by *trans*-differentiation from differentiated cells at apoplasmic/symplasmic boundaries in nutrient transport. Adaxial epidermal cells of isolated cotyledons can be induced to form functional TCs, thus providing a valuable experimental system to investigate genetic regulation of TC *trans*-differentiation. The genome of *V. faba* is exceedingly large (ca. 13 Gb), however, and limited genomic information is available for this species. To provide a resource for future transcript profiling of epidermal TC differentiation, we have undertaken *de novo* assembly of a genome-wide transcriptome map for *V. faba*. Illumina paired-end sequencing of total RNA pooled from different tissues and different stages, including isolated cotyledons induced to form epidermal TCs, generated 69.5 M reads, of which 65.8 M were used for assembly following trimming and quality control. Assembly using a De-Bruijn graph-based approach generated 21,297 contigs, of which 80.6% were successfully annotated against GO terms. The assembly was validated against known *V. faba* cDNAs held in GenBank, including transcripts previously identified as being specifically expressed in epidermal cells across TC *trans*-differentiation. This genome-wide transcriptome map therefore provides a valuable tool for future transcript profiling of epidermal TC *trans*-differentiation, and also enriches the genetic resources available for this important legume crop species.

## Introduction

The introduction of RNA sequencing (RNA-Seq) has widened the use of plant species for molecular genetic analyses beyond those for which full genome sequences are available. *De novo* assembly of transcriptomes from such species has enabled gene discovery and marker identification in a wide variety of non-genome-sequenced agricultural species such as common wheat (Duan et al., 2012), peanut (Zhang et al., 2012), and rubber tree (Mantello et al., 2014), but especially in crop legumes such as pea (Franssen et al., 2011), yellow lupin (Parra-González et al., 2012), lentils (Verma et al., 2013), and chick pea (Garg et al., 2011a,b), which are important to agriculture but genetic studies have been limited due to their often large genomes. In combination with *de novo* assembly of novel transcriptomes, RNA-Seq provides a platform for transcript profiling in these non-annotated species, thus enabling a deeper understanding at the transcriptional level of diverse but little studied biological processes in plants, such as fiber development in ramie (*Boehmeria nivea* L.; Chen et al., 2014), cell wall synthesizing enzymes in paper mulberry (Xianjun et al., 2014), biomass production in *Panicum hallii* (Meyer et al., 2012), and plant sex determination in cucumber (Guo et al., 2010).

*Vicia faba* (L.) is an important cool-season grain legume species used widely for human nutrition, fodder for livestock and as a rotation crop for nitrogen replenishment of soils (Torres et al., 1993; El-Rodeny et al., 2014). In addition to its importance as a crop species worldwide, *V. faba* is also widely used in plant physiology research to study such processes as guard cell dynamics in leaves (Fukuda et al., 1998; Fellè et al., 2000), transport processes in phloem (Thorpe et al., 2010; Hafke et al., 2013), and importantly for this study, transfer cell (TC) development in cotyledons (Offler et al., 2003). In the latter case, when isolated cotyledons are placed in culture, adaxial epidermal cells *trans*-differentiate to become functional epidermal TCs (Farley et al., 2000; Dibley et al., 2009). This cotyledon culture system has been used extensively to investigate the development and composition of the reticulate wall ingrowth network that defines TC identity in these cells (Talbot et al., 2001, 2007a,b; Vaughn et al., 2007), in addition to defining the role of inductive signals such as sugars (Wardini et al., 2007), auxin (Dibley et al., 2009), ethylene (Zhou et al., 2010; Andriunas et al., 2011), reactive oxygen species (Andriunas et al., 2012; Xia et al., 2012), and calcium (Zhang et al., 2015) in polarized wall ingrowth deposition. In an initial study to analyse transcriptional changes occurring across induction and development of adaxial epidermal TCs in cultured cotyledons, Dibley et al. (2009) used cDNA-amplified fragment length polymorphism (AFLP) to detect 5795 transcript-derived fragments (TDFs), of which 264 showed epidermal-specific changes in expression. However, due to the limited capacity of cDNA-AFLP for high-throughput analysis, only 112 TDFs from this cohort of 264 were sequenced to provide a glimpse of the diversity of genes involved in signaling, metabolism, cell division, vesicle trafficking, and cell wall biosynthesis putatively involved in TC *trans*-differentiation (Dibley et al., 2009). Importantly, however, this study estimated that TC formation in this system may involve differential expression of approximately 650 different genes (Dibley et al., 2009), a total approaching the minimum 815 genes determined from a 12 K microarray analysis to be differentially expressed during nucellar projection and endosperm TC development in barley grains (Thiel et al., 2008).

RNA-Seq provides an attractive technical platform for deep transcript profiling of the *trans*-differentiation of epidermal TCs in *V. faba* cotyledons. However, the genome size of *V. faba* is estimated to be 13 Gb (Kaur et al., 2012), one of the largest described to date for crop legumes, thus making this species an unattractive target for genome sequencing but nonetheless compatible for *de novo* transcriptome assembly (Kaur et al., 2012). To provide a platform for future RNA-Seq analysis of gene expression during TC differentiation, in this communication we report the *de novo* assembly of a genome-wide transcriptome map of *V. faba* using Illumina-based 100 bp paired-end sequencing. Total RNA was isolated from diverse tissues and organs of *V. faba*, including isolated cotyledons induced to form epidermal TCs. Therefore, this genome-wide transcriptome database would be expected to contain genes involved in the *trans*-differentiation of epidermal TCs in *V. faba* cotyledons, and thus provide a reference transcriptome map suitable for transcript profiling of this process by RNA-Seq.

## Materials and methods

### Plant growth and harvesting

*Vicia faba* L. (cv. Fiord) plants were grown in environmentally-controlled glasshouse conditions as previously described (Farley et al., 2000). To survey *V. faba* transcripts, selected tissues were harvested from flowering plants including expanding and fully expanded leaves, elongating and fully elongated stems, and closed and open flowers. Whole roots including root hairs were isolated from 10-day old seedlings germinated on filter paper. At least three independent biological samples of each tissue type were harvested and snap frozen in liquid nitrogen.

To enrich the transcriptome with genes involved in induction and wall ingrowth deposition in epidermal TCs, isolated cotyledons cultured for 0, 3, 9, and 24 h were also sampled. For this process, *V. faba* cotyledons (100–120 mg FW) were removed from pods and either fixed immediately (*t* = 0 h) in ice-cold ethanol:acetic acid (3:1 v/v) for 1 h, or placed adaxial surface down on filter paper soaked in Murashige and Skoog medium (MS; Sigma Australia) in Petri dishes (Dibley et al., 2009). The Petri dishes were sealed with tape and incubated in darkness at 22°C for 3, 9, or 24 h, then the cotyledons were fixed in ice-cold ethanol:acetic acid as described above. Fixation of cotyledons in this manner was used to rapidly inhibit RNA degradation (see Dibley et al., 2009). Fixed cotyledons were rinsed briefly in distilled water, snap frozen in liquid nitrogen and stored at −80°C before isolation of total RNA. A minimum of 3 cotyledons derived from separate pods were sampled for each time point for this analysis.

### Total RNA isolation and cDNA library preparation

Total RNA was independently extracted from each batch of isolated organs/tissues (harvested as described above) using an RNeasy Plant RNA isolation kit (Qiagen) incorporating on-column DNA digestion. For transcriptome assembly, up to 1 μg of total RNA from each organ/tissue sample type was pooled to achieve 20 μg in total, of which at least 4 μg was derived from cultured cotyledon tissue. The pooled total RNA was subjected to QA analysis using an Agilent 2100 Bioanalyzer. A single cDNA library for 100-bp paired-end sequencing was constructed from this pooled total RNA using the Illumina TrySeq Library kit and sequencing was performed using the Illumina HiSeq-2000 platform. QA analysis, library construction, and sequencing were performed by the Beijing Genome Institute (Hong Kong).

### *De novo* assembly of contigs

Illumina sequencing generated 69,543,694 100-bp paired-end reads from the pooled total RNA used for transcriptome assembly. Sequence filtering was performed by trimming adapter sequences, excluding low quality reads, and ribosomal-derived reads and removing the last 10 bases from the 3′ end of each read to increase sequence confidence. The resulting 65,795,198 90-bp reads were then assembled using a De-Bruijn graph-based *de novo* assembly program in CLC Genomics Workbench 6.1 run on an Intel® Xeon® workstation with 64 Gb RAM. Word size (*k*-mer) and bubble size were varied to obtain optimum *de novo* assembled contigs (Henkel et al., 2012), based on assembly output parameters such as high N50, low total number of contigs, high average contig length, and high percentage of mapped reads (Garg et al., 2011a; Annadurai et al., 2012).

### Validation of *de novo* assembled contigs

The optimum assembly (*47*×*300*—see Results) derived from comparing *k*-mer and bubble size was used to create a BLAST database in CLC Genomics Workbench 6.1. Contig validation using 32 full-length *V. faba* cDNA sequences from GenBank was then performed by executing a BLASTN search for each cDNA against the *47*×*300* assembly and choosing the top two contigs with highest bit scores (Mizrachi et al., 2010). The outcome of BLASTN alignment parameters for each of the 32 cDNAs with their best hit contig was assessed for validating the contigs derived from the *47*×*300* assembly.

### Functional annotation

Contigs derived from the *47*×*300* assembly were functionally annotated by BLASTX analysis against both the Viridiplantae and *Arabidopsis* databases using default parameters in CLC Genomics Workbench 6.1. The BLASTX output files from both searches were then used to assign the three GO terms of Biological Process (BP), Molecular Function (MF), and Cellular Component (CC) separately using the Blast2GO plugin in CLC Genomics Workbench 6.1 (Conesa et al., 2005). The annotations from both datasets were merged to yield at least one annotation for most of the contigs. The “GO slim” routine in Blast2GO was performed on the merged annotation dataset in order to merge specific GO terms into higher-order generic terms for each contig. This merging eases the process of creating a “combined graph,” which is a directed acrylic graph that summarizes the functional relationship within and between all three GO terms (BP, MF, CC) using Blast2GO (Conesa et al., 2005; Gotz et al., 2008).

## Results

### Generation of a *de novo*-assembled, genome-wide transcriptome map of *V. faba*

**Figure 1:**
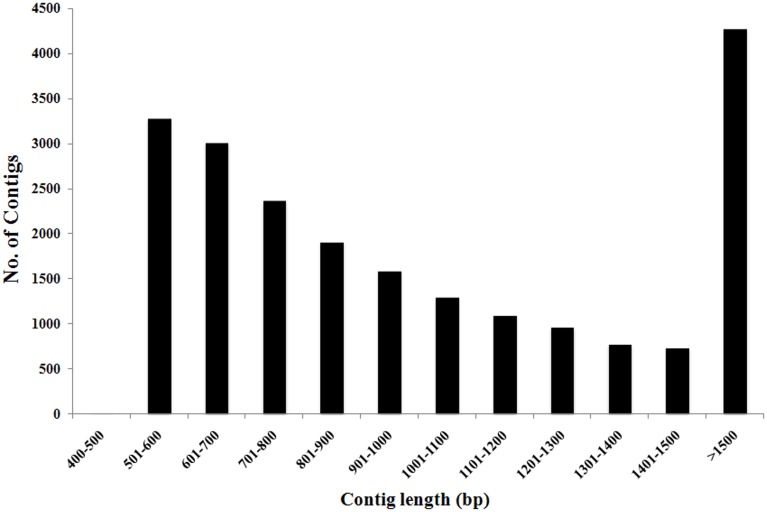
**Distribution of total number of *V. faba* contigs vs. contig length**. Most contig lengths were in the range of 500–800 bp (50%) and above 1500 bp (20%). Total number of contigs decreased as contig length increased.

To construct a genome-wide transcriptome map of *V. faba*, total RNA was isolated from all major organs across different stages of development, including isolated cotyledons cultured for 0, 3, 9, and 24 h (see Materials and Methods). Illumina sequencing of this pooled total RNA yielded approximately 69.5 M paired-end reads, and after sequence filtering and quality control, a total of nearly 65.8 M 90-bp paired-end reads were used for *de novo* assembly using CLC Genomics Workbench 6.1 (see Materials and Methods). *De novo* assembly was optimized by comparing word size (*k*-mer) and bubble size on assembly outcomes (Henkel et al., 2012). From 24 different assemblies (see Supplementary Table 1), assembly *47*×*300* (*k*-mer 47, bubble size 300) was chosen based on the maximum number of optimum parameters such as high N50 value (1245), low total number of contigs (21,297), high values for both longest contig length (10,528), and percentage of mapped reads (82%) contributing to the assembly (see Table 1; Garg et al., 2011a; Annadurai et al., 2012; Henkel et al., 2012; Dorn et al., 2013). The parameter Reads Mapped Back to Transcripts (RMBT) is an indication of completeness of assembly for *de novo* assembled transcripts (Moreton et al., 2014), and assembly *47*×*300* returned a high score of 82% (see Table 1). This assembly was chosen in preference to assembly *20×300* due to the latter's low value for percentage mapped reads (see Supplementary Table 1). Furthermore, a *k*-mer value of 47 is consistent with previous studies using this value as an optimum word size for *de novo* transcriptome assembly in other non-sequenced species such as chick pea (*Cicer arietinum*; Garg et al., 2011a) and insulin plant (*Costus pictus*; Annadurai et al., 2012). Contig length in assembly *47*×*300* varied from 432 bp to 10,528 bp, with an average of 1114 bp. Total number of contigs decreased with increasing contig length, with the majority of assembled contigs belonging to the size range 500–800 bp and >1500 bp (see Figure 1), while total reads mapped per contig ranged from 33 to 1,015,710 with an average of 2528 (see Table 2). A majority of the reads were mapped to assembled contigs greater than 1500 bp length, with the least amount of reads mapping to contigs of 400–600 bp (see Table 2). The contigs making up assembly *47*×*300* have been submitted to the Cool Season Food Legume Genome Database (www.coolseasonfoodlegume.org), while the 69.5 M paired-end raw reads have been submitted to Short Read Archive database (NCBI; accession number SRP055969).

**Table 1:** **Statistics of RNA-Seq and *de novo* assembly (*47*×*300*) of *Vicia faba* transcriptome map**.

Total number of filtered reads	65,795,198
Reads used for assembling contigs	53,838,233
Total number of contigs	21,297
N50 (bp)	1245
Average contig length (bp)	1114
Longest contig length (bp)	10,528
Mapped reads (%)	82
Average number of reads per contig	2528

**Table 2:** **Statistics of mapped reads assembled into contigs in assembly *47*×*300***.

**Contig length (bp)**	**Average total read count**	**Total read count**	
		**Lowest**	**Highest**
400–500	546	55	1633
501–600	803	33	401,177
601–700	1471	39	883,766
701–800	1289	53	135,973
801–900	1774	62	320,721
901–1000	1759	79	381,139
1001–1100	2288	101	425,383
1101–1200	2229	104	74,043
1201–1300	2988	162	552,613
1301–1400	2642	196	185,390
1401–1500	3371	207	502,245
>1500	5793	172	1,015,710

### Validation of the genome-wide transcriptome map

To assess the validity of assembly *47*×*300*, 32 full-length *V. faba* cDNAs available in GenBank were compared by BLASTN against the 21,297 contigs that make up assembly *47×300*. Of this cDNA collection, ranging in length from 259 to 3426 bp, 29 cDNAs returned matches of 97–100% identity, while only three returned matches between 90 and 94% (see Table 3). This result indicates that the assembled contig closely matched the relevant cDNA across the length of the transcript-derived contig. Several of the cDNAs that best aligned to contigs in assembly *47*×*300* corresponded to *V. faba* cDNAs previously shown to have putative roles in signaling epidermal TC *trans*-differentiation (see Table 3; Zhou et al., 2010; Andriunas et al., 2012). For example, three ethylene response factor genes (*VfERF1-3*; Zhou et al., 2010), and two putative respiratory burst oxidase homolog genes (*VfrbohA, VfrbohC*; Andriunas et al., 2012), each of which shows epidermal-specific change in expression accompanying the *trans*-differentiation of epidermal TCs, all matched with 98–100% identity across corresponding contig sequence coverage present in assembly *47*×*300* (see Table 3).

**Table 3:** **BLASTN output for 32 full-length *Vicia faba* cDNAs assessed against assembly *47*×*300***.

**GeneBank entry (Accession number)**	**Hit length (bp)**	**Query length (bp)**	**Length coverage (%)**	**Identity (%)**
UDP glucose D fructose 2 glycosyl transferase mRNA (M97551)	1721	2665	64	99
VHA2 mRNA for P-type H^+^-ATPase (AB022442)	3277	3398	96	99
Glutamate decarboxylase (JX444699)	1625	1787	91	99
Putative ABA induced guard cell protein ABG1 mRNA (AF218806)	648	753	86	99
Pre-pro cysteine proteinase VFCYSPRO mRNA (U59465)	1435	1439	100	99
GPRP mRNA for glycine- and proline-rich protein (AB615379)	850	860	99	99
NOI mRNA for putative nitrate induced NOI protein (AB615378)	255	259	98	100
Calcium dependent protein kinase 1 CPK1 mRNA (AY753552)	993	1783	56	100
Cyclophillin (L32095)	1101	1160	95	99
CBL1 mRNA calcineurin-B like calcium binding protein (AB370168)	722	1007	72	100
CIPK1 mRNA for CBL interacting protein (AB370167)	1045	1400	75	99
Peptide transporter 1 PTR1 mRNA (AY289622)	1438	2028	71	99
Heat shock protein 17.9 gene (KC249973)	472	483	98	90
Putative glycerol-3-phosphate acyltransferase GPAT mRNA (AF090734)	1385	1386	100	99
Ferredoxin NADP reductase precursor fnr mRNA (U14956)	1455	1474	99	98
Putative ethylene responsive factor ERF3 mRNA^*^ (EU543661)	671	672	100	100
Putative ethylene responsive factor ERF2 mRNA^*^ (EU543660)	670	1232	54	98
Putative ethylene responsive factor ERF1 mRNA^*^ (EU543659)	654	688	95	100
Putative ethylene insensitive transcription factor (EIN3-1) mRNA^*^ (EU543660)	2173	2406	90	99
Putative ethylene insensitive transcription factor EIN3-2 mRNA^*^ (EU543658)	2296	2353	97	100
Putative respiratory burst oxidase-like protein A (rbohA) mRNA^*^ (JF784279)	2824	3046	93	100
Putative respiratory burst oxidase-like protein C (rbohC) mRNA^*^ (JF784280)	1121	3426	33	99
Putative ACC synthase ACS2 mRNA^*^ (EU543656)	964	1519	63	97
Putative aminocyclopropane carboxylic acid oxidase (ACO1)^*^ (EU543653)	614	614	100	99
Putative aminocyclopropane carboxylic acid synthase (ACO2)^*^ (EU543654)	764	767	100	100
Apyrase gene (AB088209)	294	2173	13	92
Expansin EXP1 mRNA (EF190969)	1037	1127	92	99
PP2Ac-3 mRNA for type 2A protein phosphatase-3 (AB039918)	1342	1502	89	99
PP2Ac-2 mRNA for type 2A protein phosphatase-2 (AB039917)	1397	1475	95	99
CyP mRNA (AB012947)	806	829	97	100
MET mRNA for type 1 metallothionein (AB176562)	345	501	69	94
Guanine nucleotide regulatory protein mRNA (Z24678)	632	946	67	100

Hit length refers to contig length homologous to V. faba cDNA sequence; Query length refers to total length of V. faba cDNA sequence. Asterisks (

* *) indicate genes previously cloned from isolated epidermal TCs of V. faba cotyledons—many of these genes are reported as showing epidermal-specific differential expression across epidermal TC trans-differentiation in cultured cotyledons (see Zhou et al., 2010; Andriunas et al., 2012)*.

### Functional annotation of contigs

**Figure 2:**
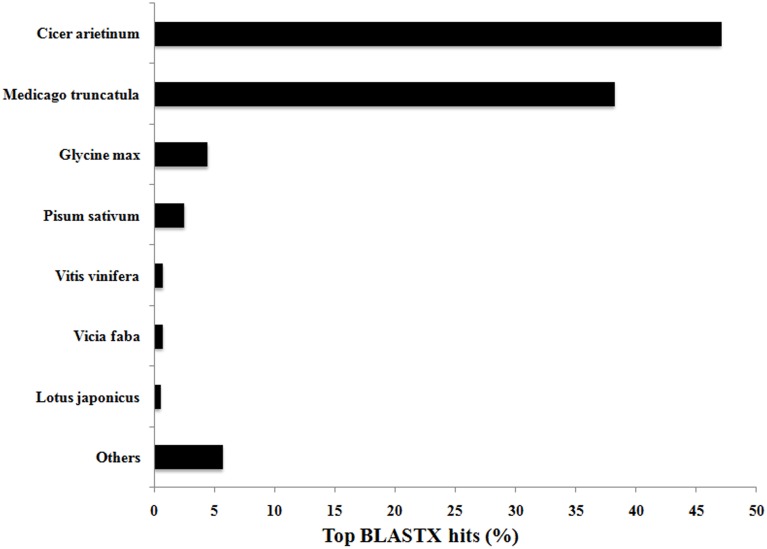
**Top BLASTX hits of *V. faba* contigs against the Viridiplantae database**. Contig sequences of *V. faba* are highly conserved with closely related and sequenced legume species, with 85% of the contigs showing similarity with *Cicer arietinum* (chick pea) and *Medicago truncatula*.

Functional annotation was undertaken to assign GO terms to the 21,297 contigs comprising assembly *47*×*300*. BLASTX analysis in CLC Genomics Workbench yielded 20,325 and 20,052 annotation hits when compared against the Viridiplantae and *Arabidopsis* data sets, respectively. The Blast2GO plugin in CLC successfully annotated 12,192 and 15,794 contigs from the Viridiplantae and *Arabidopsis* datasets, respectively, and thus merging these two annotation datasets (Garg et al., 2011a) yielded at least one annotation term for 17,160 (80.6%) of the 21,297 contigs in *47*×*300*. This total of 80.6% is comparable to annotation statistics for *de novo* assemblies from other non-sequenced species, or yet to be sequenced at the time of experimentation, such as chick pea (85.5%; Garg et al., 2011a), eucalyptus (*Eucalyptus grandis*—83.2%; Mizrachi et al., 2010), insulin plant (69.2%; Annadurai et al., 2012), sesame (*Sesamum indicum*—54.0%; Wei et al., 2011), and sweet potato (*Ipomoea batatas—*62.0%; Wang et al., 2010). As expected, *V. faba* transcripts compared against the Viridiplantae database by BLASTX showed highest similarity to closely related legume species for which full genome sequences are now available, with *Cicer arietinum* (chick pea) and *Medicago truncatula* genes accounting for 47% and 38% of the annotated hits, respectively (see Figure 2).

**Figure 3:**
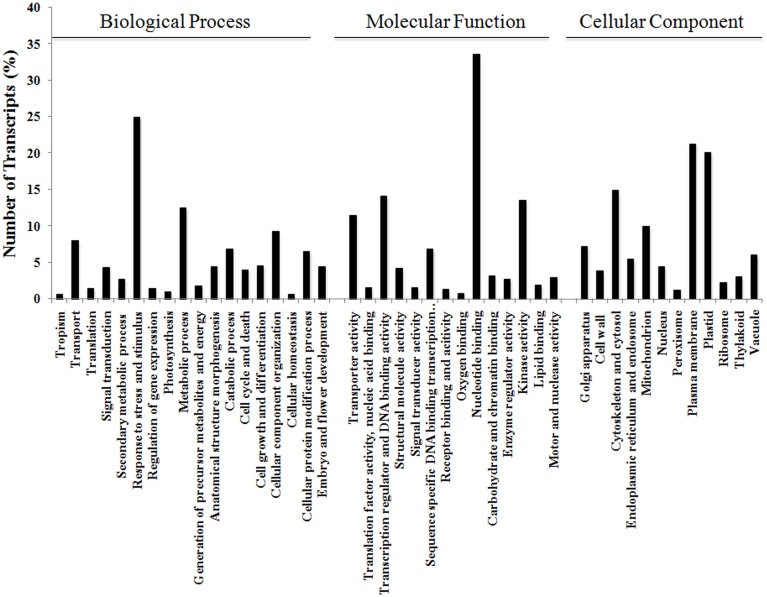
**Functional annotation of *V. faba* contigs**. Blast2GO characterization of GO terms in the three categories of Biological Process, Molecular Function, and Cellular Component.

The 17,160 annotated contigs (herein referred to as unigenes) were assigned GO terms in three categories, namely Biological Process, Molecular Function, and Cellular Component (see Figure 3). Within Biological Process, the category “Response to different stress and stimulus,” which incorporates biotic, abiotic, endogenous, and extracellular, was highly represented (25%) followed by “Metabolic process” (13%). Within Molecular Function, processes like “Nucleotide binding” (34%), “Transcriptional regulation and DNA binding” (14%), and “Kinase activity” (14%) were dominant in this category. Similarly, Cellular Component was highly represented by “Plasma membrane” (21%) followed by “Plastid” (20%) subgroups.

**Figure 4:**
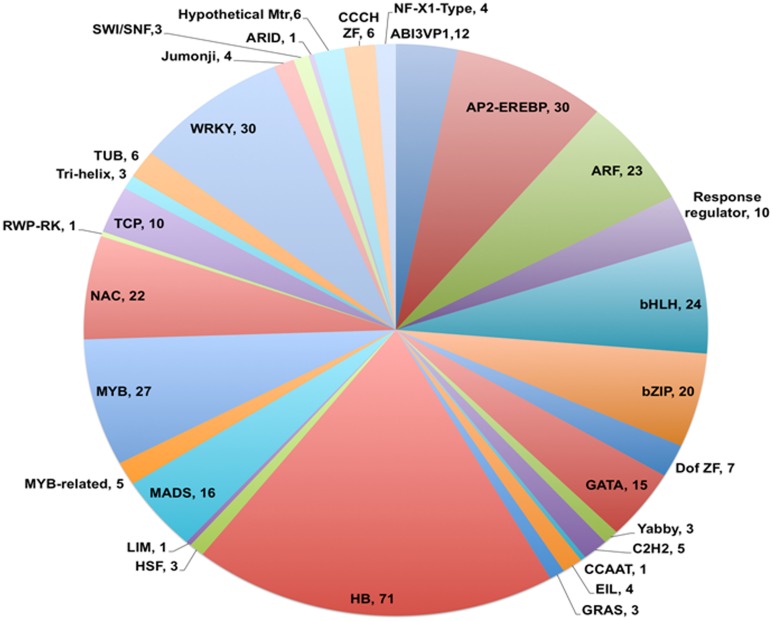
**Distribution of *V. faba* transcripts into different transcription factor families**. Pie chart showing families of transcription factors and total number of members identified for each family. Homeobox family members (HB) contributed 19% of the total transcription factors identified and classified, followed by WRKY (8%) and AP2-EREBP (8%) sequences. The number listed after each family name represents the total number of transcription factors assigned to that family.

Analysis of the annotated data set showed that 726 unigenes (4.2% of 17,160 in total) were identified as coding for putative transcription factors based on the GO term 0003700 (Transcription factor activity) appearing in the output for Molecular Function category. Of this total, 376 could be assigned to 31 of the 58 families of known plant transcription factors identified in PlnTFDB based on presence of family name in the annotation (see Figure 4; Perez-Rodriguez et al., 2009). A similar representation of plant transcription factor families has been observed in *de novo* assembled transcriptomes from chick pea (Garg et al., 2011b), soybean (Schmutz et al., 2010), and other legumes (Libault et al., 2009). Of those unigenes assigned to the 31 families, 19% were classified as homeobox transcription factors, with WRKY and AP2-EREBP genes making up the next most prominent families at 8% each (Figure 4).

## Discussion

We have generated a genome-wide transcriptome map of *V. faba* to provide a reference for future RNA-Seq-based transcript profiling of TC development. Despite the large genome size of this important crop legume (ca. 13 Gb; Kaur et al., 2012), the paired-end strategy using Illumina sequencing was successful in generating 21,297 contigs, of which 80.6% (17,160) could be annotated against GO terminology. The total number of gene loci in *V. faba* is not known, but the 17,160 unigenes identified in this study may represent at least half of the gene space in faba bean given the number of genes identified in flowering plants (~27,000–39,000; TAIR10—www.arabidopsis.org; Rice Genome Annotation Project—http://rice.plantbiology.msu.edu/). This argument is based in part on a similar study by Lehnert and Walbot (2014) who reported that *de novo* assembly of 20,881 contigs from *Dahlia* may represent about half the gene loci in this species, which has a genome of approximately 9.4 Gb.

Validation of the *47*×*300* assembly against full-length *V. faba* cDNAs in GenBank established that the CLC assembly routine using a De-Bruijn graph-based approach gave 97–100% identity for 29 out of the 32 cDNAs tested across an average “Hit length” of 1157 bp, thus providing high confidence that the *47*×*300* assembly accurately reflects the component of the *V. faba* transcriptome captured by the sequencing strategy. Furthermore, the value of the transcriptome map as a resource for future transcript profiling of TC *trans*-differentiation is indicated by the presence of several transcripts, such as three ethylene response factor genes (*VfERF1-3*), two respiratory burst oxidase homologs (*VfrbohA*, *VfrbohC*), two ethylene insensitive transcription factors (*VfEIN3-1*, *VfEIN3-2*), two putative ACC oxidase genes (*VfACO1*, *VfACO2*, and two putative ACC synthase genes (*VfACS1*, *VfACS1*). Each of these genes has been cloned from isolated epidermal tissue, with many displaying epidermal-specific differential expression during the *trans*-differentiation of epidermal TCs (Zhou et al., 2010; Andriunas et al., 2012).

A major aim of our future investigations is to identify transcription factors regulating the *trans*-differentiation of TCs. Annotation of the *47*×*300* assembly revealed that 4.2% (726) of the 17,160 unigenes encoded putative transcription factors, of which representatives from 31 of the 58 known families of transcription factors in plants were identified. Within this cohort, 19% were identified as homeobox genes, while WRKY and AP2-EREBP members were the next most prominently represented families at 8% each. Homeobox genes are commonly involved in regulating morphogenesis and cell identity, typically via switching on cascades of gene expression (Williams, 1998). WRKY transcription factors are involved in biotic and abiotic stress defense pathways in plants (Rushton et al., 2010), and members of the AP2-EREBP family have roles in stress signaling pathways induced by hormones like ethylene and methyl jasmonate (Dietz et al., 2010). Given that the *trans*-differentiation of TCs involves differential expression of many hundreds of genes (Thiel et al., 2008; Dibley et al., 2009) in response to biotic (Cabrera et al., 2014) and abiotic stresses that can involve ethylene (Zhou et al., 2010) and methyl jasmonate (Amiard et al., 2007), the diverse representation of transcription factors in this transcriptome map augers well as a reference tool to identify key transcriptional regulators of TC *trans*-differentiation.

Overall, these considerations support the conclusion that assembly *47*×*300* represents a high-quality, genome-wide transcriptome map of *V. faba* and thus provides a validated platform for subsequent transcript profiling of TC *trans*-differentiation by RNA-Seq.

### Conflict of interest statement

The authors declare that the research was conducted in the absence of any commercial or financial relationships that could be construed as a potential conflict of interest.
