# Supplementary material for: De novo assembly of a genome-wide transcriptome map of Vicia faba (L.) for transfer cell research
Source: Front Plant Sci. 2015 Apr 9;6:217. doi: 10.3389/fpls.2015.00217 (PMC4391045; doi:10.3389/fpls.2015.00217)
Supplement: Supplementary file 1 [file Table1.DOCX]

***Supplementary Material***

***De novo* assembly of a genome-wide transcriptome map of *Vicia faba* (L.) for transfer cell research**

*Kiruba S. Arun-Chinnappa and David W. McCurdy**

*Centre for Plant Science, School of Environmental and Life Sciences, The University of Newcastle, Callaghan, NSW 2308, Australia.*

****Correspondence:*** *David W. McCurdy, School of Environmental and Life Sciences, The University of Newcastle, University Drive, Callaghan, NSW 2308, Australia*

**Supplementary Table 1. *De novo* assembly outputs resulting from varying bubble size and word size.**

| **WORD SIZE**  **(*k*-mer)** | **BUBBLE SIZE** | **N50**  **(bp)** | **TOTAL No. OF CONTIGS** | **LARGEST CONTIG LENGTH**  **(bp)** | **AVERAGE CONTIG LENGTH**  **(bp)** | **MAPPED READS**  **%** |
| --- | --- | --- | --- | --- | --- | --- |
| 20 | 200 | 1527** | 22066 | 11541* | 1299 | 75 |
| 20 | 300 | 1577* | 19153* | 10589 | 1320* | 65 |
| 23 | 200 | 1518 | 21706 | 9455 | 1291 | 79 |
| 23 | 300 | 1521 | 21680 | 9456 | 1293** | 80 |
| 25 | 200 | 1512 | 22052 | 9700 | 1289 | 78 |
| 25 | 300 | 1512 | 22020 | 9702 | 1290 | 76 |
| 27 | 200 | 1499 | 22397 | 9646 | 1276 | 77 |
| 27 | 300 | 1501 | 22402 | 9646 | 1275 | 77 |
| 30 | 200 | 1467 | 22563 | 9478 | 1256 | 84* |
| 30 | 300 | 1466 | 22541 | 9481 | 1255 | 81 |
| 33 | 200 | 1447 | 22429 | 9304 | 1243 | 78 |
| 33 | 300 | 1452 | 22429 | 9305 | 1247 | 79 |
| 35 | 200 | 1423 | 22444 | 9916 | 1228 | 79 |
| 35 | 300 | 1426 | 22447 | 9916 | 1229 | 80 |
| 37 | 200 | 1394 | 22314 | 9304 | 1208 | 79 |
| 37 | 300 | 1396 | 22298 | 9304 | 1209 | 80 |
| 40 | 200 | 1364 | 22120 | 11455** | 1190 | 81 |
| 40 | 300 | 1352 | 22079 | 11455 | 1184 | 81 |
| 43 | 200 | 1324 | 21884 | 10668 | 1160 | 80 |
| 43 | 300 | 1326 | 21868 | 10668 | 1162 | 80 |
| 45 | 200 | 1282 | 21697 | 10621 | 1132 | 81 |
| 45 | 300 | 1283 | 21709 | 10621 | 1133 | 81 |
| 47 | 200 | 1245 | 21295** | 10528 | 1114 | 82** |
| 47 | 300 | 1245 | 21297** | 10528 | 1114 | 82** |

Single asterisk (*) indicates best score and double asterisk (**) indicates second best score, for each criteria as follows: highest N50 score, lowest number of contigs, largest contig length of all assemblies, average length of contigs (includes all contigs in the assembly), highest mapped reads %.
